# Skeletal Muscle Regeneration on Protein-Grafted and Microchannel-Patterned Scaffold for Hypopharyngeal Tissue Engineering

**DOI:** 10.1155/2013/146953

**Published:** 2013-09-23

**Authors:** Zhisen Shen, Shanshan Guo, Dong Ye, Jingjing Chen, Cheng Kang, Shejie Qiu, Dakai Lu, Qun Li, Kunjie Xu, Jingjing Lv, Yabin Zhu

**Affiliations:** ^1^Department of Otorhinolaryngology of Lihuili Hospital, Ningbo University, Ningbo 315211, China; ^2^The Medical School, Ningbo University, Ningbo 315211, China

## Abstract

In the field of tissue engineering, polymeric materials with high biocompatibility like polylactic acid and polyglycolic acid have been widely used for fabricating living constructs. For hypopharynx tissue engineering, skeletal muscle is one important functional part of the whole organ, which assembles the unidirectionally aligned myotubes. In this study, a polyurethane (PU) scaffold with microchannel patterns was used to provide aligning guidance for the seeded human myoblasts. Due to the low hydrophilicity of PU, the scaffold was grafted with silk fibroin (PU-SF) or gelatin (PU-Gel) to improve its cell adhesion properties. Scaffolds were observed to degrade slowly over time, and their mechanical properties and hydrophilicities were improved through the surface grafting. Also, the myoblasts seeded on PU-SF had the higher proliferative rate and better differentiation compared with those on the control or PU-Gel. Our results demonstrate that polyurethane scaffolds seeded with myoblasts hold promise to guide hypopharynx muscle regeneration.

## 1. Introduction

Hypopharynx carcinoma is one of the common head and neck cancers; approximately 2500 new cases are diagnosed in the United States each year with the peak incidence in males and females aged 50 to 60 years [1, 2]. Laryngopharyngectomy and reconstruction followed by chemoradiotherapy has been the traditional and relatively practicable treatment presently. However, surgical interventions are inevitably associated with quality-of-life impairments including severe speech and swallowing disability [3]. Also, physical deformities and emotional trauma persist long after the conclusion of surgical intervention. For the severe and large area hypopharynx defects, visceral or myocutaneous flaps, such as an ileocolic flap, a radial forearm flap, and an anterolateral thigh flap, a submental island flap, and an infrahyoid myocutaneous flap, are common methods used for surgical repair [4–8]. However, these grafts cannot fully restore the function of hypopharynx tissue, in particular the reconstruction of the complex structures surrounding the larynx and hypopharynx, while postoperative dyspnea may be inevitable when hypopharynx pharyngeal constrictors were sectioned [9, 10].

Skeletal muscle is the most abundant tissue type in the human body. Muscle fiber consists of a longitudinal arrangement of myofilaments with actin and myosin as major components [11]. Huang and colleagues cultured murine myoblasts (ATCC C2C12) on elastic poly(dimethylsiloxane) (PDMS) films topographically micropatterned with 10 *μ*m wide microgrooves. The myoblasts formed long and unbranched myotubes that had uniform diameter and were aligned parallel to the microgroove direction to suggest that microgrooves promote end to end fusion of myoblasts [12]. In another study by Ma and coworkers, ATCC were seeded onto a collagen composite scaffold and cultured in a roller bottle cell culture system to create a 3-dimensional (3D) tissue graft *in vitro*. The 3D graft was then used for *in vivo* muscle tissue repair by implanting it into defect sites created in mice models. The scaffolds were found to degrade slowly over time, and muscle healing was improved as shown by an increased quantity of innervated and vascularized muscle fibers. These results suggest that 3D muscle grafts created *in vitro* from collagen composite scaffolds seeded with myoblasts can be used for defect muscle tissue repair *in vivo* [13].

In our experiment, scaffolds with linearly aligned microchannels were fabricated to align the seeded myoblasts through contact guidance. Protein grafted surfaces promote human hypopharyngeal cell proliferation and differentiation. Furthermore, we will offer a new perspective on the implications of *ex vivo* niche system to advance the scientific understanding of stem cell functions as well as supporting clinical applications.

## 2. Materials and Methods

### 2.1. Materials

Poly(ester urethane) (PU, 58213 NAT 022) was purchased from Estane Co., China. Dulbecco's modified Eagle's medium (DMEM), fetal bovine serum (FBS), antibiotic antimycotic solution (AAS, 100 U/mL penicillin, 100 *μ*g/mL streptomycin, and 100 *μ*g/mL amphotericin B), and Trypsin-EDTA solution were purchased from Gibco (Invitrogen, Co., New York). Anti-MyoD1 antibody was supplied by Abcam (Hong Kong) Ltd. FITC-conjugated AffiniPure goat anti-mouse IgG (H+L) was purchased from Jackson ImmunoResearch. Rabbit anti-beta-actin (loading control), HRP-conjugated goat anti-rabbit IgG (H+L) and HRP-conjugated goat anti-rat IgG (H+L), were supplied by Beijing Biosynthesis Biotechnology Co., Ltd. All chemical reagents used for Western blotting were purchased from Beyotime Institute of Biotech (JiangSu, China). Other chemical reagents used in this experiment were from Sinopharm Chemical Reagent. All water used in the experiment was doubly distilled. Phosphate buffer saline (PBS, pH = 7.4) used in cell culture was sterilized.

### 2.2. Extraction of Silk Fibroin (SF)

Silk fibroin was extracted from the natural silkworm cocoon (Zhejiang Province, China) using a method reported previously [14]. The cocoons were heated to boil for 1 h in an aqueous Na_2_CO_3_ solution (0.5 wt%) and rinsed with water to remove the sericin. The extracted fibers were subsequently dissolved in calcium nitrate tetrahydrate (Ca(NO_3_)_2_·4H_2_O) at 80°C to yield a homogeneous solution (5%, w/w). The solution was then dialyzed using a tubular cellulose membrane (MW cutoff, 12,000–14,000, Sigma) in water for 3 days at room temperature and thereafter replenished with water every 4 hours (a total about 12 to 16 times) to remove the residual salts. The dialyzed solution was subsequently collected, and the SF concentration was measured to be 0.018 g/mL.

### 2.3. Scaffold Preparation

Based on our previous work, a soft polydimethylsiloxane (PDMS) mould was fabricated from a silica wafer patterned with unidirectional microchannels of 200 *μ*m in width and separated by walls with 30 *μ*m wide and 30 *μ*m high. PU was dissolved in 1,4-dioxane to get a concentration of 12% (w/v). PU/1,4-dioxane solution was casted onto the PDMS mould and dried at 37°C for 12 h, yielding a transparent PU membrane with parallel micro-channels of 200 *μ*m width and 30 *μ*m depth. The membrane was then immersed in alcohol solution (95 v%) for 3 h to remove any dirt and rinsed repeatedly with large amounts of water and dried in air.

To immobilize proteins onto the PU surface, the patterned PU membrane was immersed in 1,6-hexanediamine/propyl alcohol solution (0.06 g/mL) for 10 min at 37°C and rinsed with water for 24 h at room temperature to remove the unreacted 1,6-hexanediamine. It was subsequently vacuum-dried at 30°C for 12 h to remove residual propyl alcohol. This aminolyzed PU membrane was soaked in aqueous glutaraldehyde solution (1.0 wt%) for 3 h at room temperature to transform amino groups into the aldehyde groups and was further rinsed with abundant water to remove superfluous glutaraldehyde. The membrane was then incubated in a gelatin/PBS solution (2 mg/mL) or a SF/PBS solution for 24 h at 4°C, respectively. The gelatin-immobilized (PU-Gel) and SF-immobilized membranes (PU-SF) were rinsed with water for at least 12 h to remove all free gelatin or SF.

For cell culture tests, scaffolds were sterilized in 75% alcohol for 4 h and rinsed with sterilized PBS to remove residual alcohol. Prior to cell seeding, the scaffolds are immersed in DMEM for 30 min and placed into 96-well tissue culture polystyrene (TCPS) plates.

### 2.4. Scaffold Characterizations

#### 2.4.1. Mechanical Property Test

Mechanical testing was performed by a linear tensile tester (Instron 3366, USA) at the stretch rate of 60 mm/min. Dumbbell-shaped scaffolds with a gauge length of 22 mm and cross-sectional area of 0.2-0.3 mm × 3.8 mm were used. The mechanical properties of PU, PU-Gel, and PU-SF scaffolds were separately tested. 

For testing the alteration of scaffolds' mechanical properties after *in vitro* degradation, the scaffolds were firstly sterilized in aqueous alcohol solution (75 v%) for 2 h and rinsed in water to remove residual alcohol. They were then incubated at 37°C in PBS (pH = 7.4) supplemented with penicillin (100 U/mL)-streptomycin (100 *μ*g/mL). The mechanical testing was performed in the same way after different degradation periods (20, 105, 145, 185, 220, and 260 days). Three repeats were conducted for each scaffold type. 

#### 2.4.2. Contact Angle Measurement

The static contact angle of each PU scaffold (planar face) was measured using a surface tension-contact angle meter (DIGIDROP, GBX, France) at ambient humidity and temperature. Drops of deionized water about 1.0 *μ*L in volume were applied.

### 2.5. Cell Culture

Skeletal muscle biopsies were obtained from hypopharyngeal constrictors of 10 patients, who underwent surgery for laryngeal or hypopharyngeal carcinoma at the Department of Otolaryngology, Head and Neck surgery, Lihuili Hospital of Medical School, Ningbo University (Ningbo, China). Only specimens which were determined to be free of cancer by the pathologist were used. Collection of biopsy samples was approved by the Ethics Review Boards of Ningbo University, and an informed consent was obtained from every patient.

The biopsy specimens stored in PBS (pH = 7.4, ice-cool) supplemented with AAS upon collection were immediately transported to our laboratory for further processing. The muscle samples were first rinsed with PBS, and adherent fat and tendons tissues were removed with ophthalmic scissors. The muscle tissues were then minced into small tissue pieces of approximately 1 × 1 × 1 mm^3^ and transferred into culture flasks (Corning, USA). Tissues were adhered to the tissue culture plate before 2 mL of culture medium containing 15% FBS and AAS was added. The medium was subsequently renewed every three days. Cells from the tissue pieces were allowed to migrate outwards and grow to be confluent in the culture plate. Cells were passaged by enzymatic treatment with 0.25% Trypsin-EDTA solution.

Cells from 2nd to 5th passages were seeded on the scaffolds at a density of 8 × 10^4^ cells/mL.

### 2.6. Mitochondrial Activity Assay

Mitochondrial activity of the cells seeded on each scaffold was assayed using the MTT method at 2, 5, and 10 days, respectively. 20 *μ*L of MTT solution (0.5 mg/mL) was added to each culture well and incubated with cultured cells for 4 h at 37°C in the dark. 150 *μ*L of dimethylsulphoxide (DMSO) was subsequently added to each well to dissolve the purple formazan crystal. Absorbance was measured at 490 nm using an ELISA reader (MaxM5, Spectra). The absorbance of DMSO without formazan crystal was used as blank reference. Data were compared between cells grown on PU, PU-Gel, and PU-SF scaffolds and TCPS. Triplicates of each sample were averaged.

### 2.7. Immunofluorescence Staining

Cells cultured on scaffolds for 10 days were fixed in 4 wt% paraformaldehyde (Sigma, USA) for 30 min and rinsed three times with PBS for 10 min each time. The samples were immersed in 0.2% Triton X-100 for 10 min, rinsed three times for 10 min each time with PBS, and then blocked in 4% goat serum for 1 h in order to minimize nonspecific binding. The entire process was carried out at room temperature. The blocking solution was drained from the samples (without washing) and incubated over night in anti-MyoD1 mouse monoclonal antibody (1 : 200 dilution in PBS) at 4°C. After rinsing with PBS three times for 10 min each time, the samples were subsequently incubated in anti-mouse IgG (H+L) secondary antibody conjugated with FITC (1 : 100 dilution in PBS) for 1 h in the dark. For nuclei observation, the samples were dipped in 4,6-diamidino-2-phenylindole dihydrochloride (DAPI) solution (Sigma, 3 *μ*g/mL in PBS) and immediately rinsed with PBS. In the stained image, the MyoD1 (myogenic differentiation antigen) displayed green fluorescence while the nuclei displayed blue fluorescence.

### 2.8. Scanning Electron Microscope (SEM) Imaging

Samples were fixed in 2.5 wt% glutaraldehyde solution for 30 min at room temperature after cells were seeded for 2, 5, and 10 days, respectively. After rinsing three times for 10 min each with water, the scaffolds were mounted on an aluminum stub and coated with gold. The cell morphology was observed using a Philips CM100 electron microscope (Philips Electronics Nederland BV, Eindhoven, Netherlands) with an accelerating voltage of 10 kV.

### 2.9. Western Blotting

Cells grown on scaffolds (24-well plates) for 2, 5 and 14 days were washed with PBS three times, 5 min for each time. 200 *μ*L of Membrane and Cytosol Protein Extraction Kit (Beyotime, China) was added to each sample for 30 min. The lysate was then collected and transferred to a microcentrifuge tube. The whole process was performed on ice. Subsequently, the lysate was centrifuged at 12000 rpm for 5 min at 4°C. The supernatant was transferred to a new tube for further analysis. About 30 *μ*g of total protein (as measured by Coomassie blue staining), each with 5 *μ*L of loading buffer (5×), was then loaded into a 12% sodium dodecyl sulfate (SDS) polyacrylamide gel. Electrophoresis was performed in electrophoretic buffer solution at 100 V for 2 h. The separated proteins on the gel were then electrically transferred onto a polyvinylidene fluoride membrane (PVDF, Roche) in transfer buffer at 4°C. After being blocked with 1% bovine serum albumin (BSA) for 1 h at room temperature, the membrane was incubated overnight in anti-MyoD1 mouse monoclonal antibody (1 : 500 dilution in BSA blocking solution) at 4°C. After three rinses of 10 min each with 0.05 v% Tween-20 in Tris-buffered saline (TBST), the membrane was incubated with horse radish peroxidase-conjugated anti-mouse antibody (1 : 5000 dilution in blocking solution; Bioss, China) for 1 h at room temperature. Enhanced chemiluminescence (ECL) Western blotting detection reagents were subsequently added to the membrane to allow detection. Membranes were then exposed in a gel imaging system (Tanon-4200SF, China) where the intensity of the detected proteins was captured. The relative levels of detected proteins were assessed by the ratio between the intensity of MyoD1 and beta-actin. Beta-actin was used to normalize the cellular protein content. The results presented were from at least three separate experiments.

### 2.10. Statistics

Data are expressed as mean ± standard deviation (SD). Statistical comparisons were made by analysis of variance (ANOVA). *t*-test was used for evaluations of differences between groups. *P*values less than 0.05 were considered to be significant.

## 3. Results and Discussion

### 3.1. Properties of the Scaffolds

**Scheme 1:**
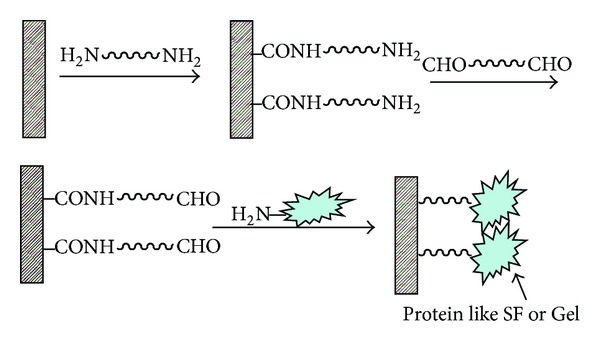
The Schematic representation of aminolysis and further immobilization of SF or Gel on PU surface. Cited from [18].

Poly(ester-urethane) is widely used as substrates in biomedical engineering due to its high mechanical elasticity, favorable hemocompatibility, and biodegradability [15]. However, its poor hydrophilicity and intrinsically inert surface result in poor cell-material interaction. In contrast, SF, a fibrous protein found in natural silkworm cocoon, has been used in a variety of biomedical applications due to its favorable mechanical properties, remarkable biocompatibility, and controllable degradation rate [16]. SF grafting is hypothesized to have potential in promoting the cytocompatibility of the scaffold to skeletal muscles. Hence, in this experiment, biomacromolecules like SF and gelatin were grafted on the surface of PU scaffolds by aminolysis and glutaraldehyde (GA) crosslinking, a method previously developed by our group [17, 18]. The grafting reaction was illustrated as Scheme 1.

**Figure 1:**
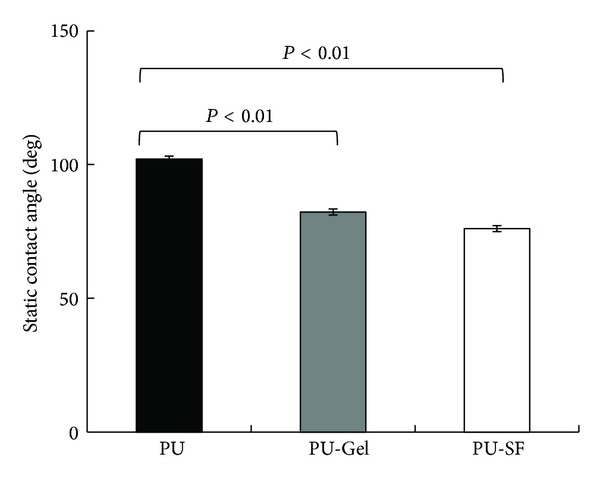
Static contact angle of water on scaffolds *P* < 0.01.

The scaffolds were tested for their wettability using static contact angle measurements (Figure 1). Following grafting, both PU-SF (76.03°  ± 2.03°; *P* < 0.01) and PU-Gel (82.27°  ± 2.24°; *P* < 0.01) scaffolds were significantly more hydrophilic than the unmodified PU scaffold (101.93°  ± 1.59°). The improvement in the wettability of the surface-modified scaffold also confirmed the success of the grafting of biomacromolecules on the PU surface.

**Figure 2:**
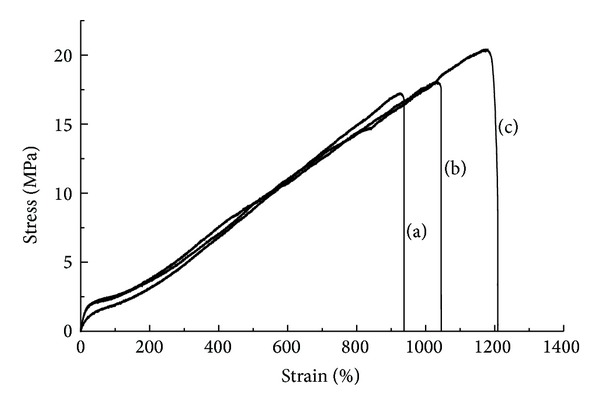
Uniaxial tensile stress strain curve of the scaffolds: (a) PU; (b) PU-Gel; (c) PU-SF.

Figure 2 shows the stress-strain behaviors of the three micropatterned scaffolds under uniaxial elongation. The PU-SF scaffold demonstrated the highest ultimate tensile strength (UTS) and maximum strain while the unmodified PU had the lowest UTS. The UTS of the PU-Gel scaffold was between that of the PU-SF and PU scaffolds. However, the moduli of all three samples were similar. As SF possesses favorable mechanical properties, the improvements in mechanical properties following the grafting of SF were expected [19], while the modulus of the surface-modified samples was predominantly determined by the intrinsic chemistry of the bulk material, that is, PU.

In tissue engineering applications, degradation is an important consideration as it is essential that the scaffold degradation characteristics match the rate of cell growth and tissue regeneration. The data obtained by *in vitro* degradation will allow us to better understand and predict the *in vivo* degradation characteristics of the scaffolds [20, 21]. We evaluated the degradation properties of PU scaffold via the measurements of weight loss and UTS over a period of 260 days. Degradation usually occurs when soluble oligomeric components diffuse and dissolve in incubation medium due to the hydrolysis of the polymeric chains [22]. The weight loss observed for all scaffolds at day 20, 105, 145, 185, 220, and 260 was very minimal, and the difference of weight loss between each sample type was minimal too. That is because the surface grafting performance shall not greatly affect the intrinsic chemistry of PU matrix. 

**Figure 3:**
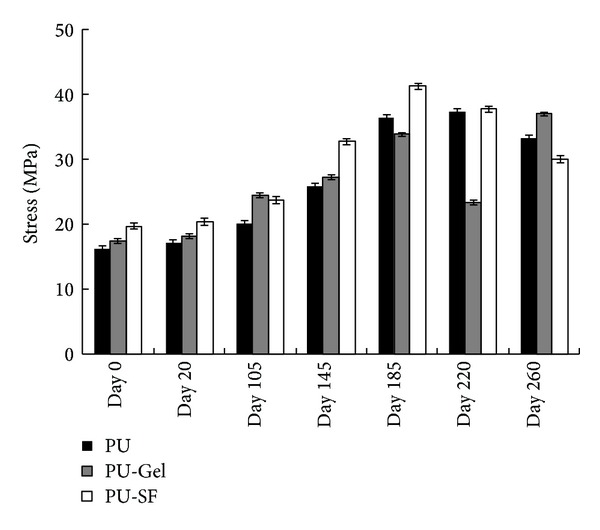
The scaffold's ultimate tensile strength (UTS) as the function of degradation time. Scaffolds were immersed in PBS (pH = 7.4) supplemented with penicillin (100 U/mL)-streptomycin (100 *μ*g/mL) at 37°C for 20, 105, 145, 185, 220, and 260 days, respectively.

On the other hand, the ultimate tensile strength (UTS) initially rises before gradually declining over time (Figure 3). Polyurethane (58213) is an aromatic polyester-based thermoplastic polymer, which is formed from the reaction of methylene diphenyl diisocyanate (MDI) and polyester polyol. This variation in mechanical properties over the course of degradation can be expected since the relative proportion of the hard segments (MDI) in PU increases due to the hydrolysis of soft segments (polyol). While the relative increase in the proportion of hard segment can enhance the UTS of PU, further increases beyond the optimal point can also adversely affect the properties of PU.

### 3.2. Aligned Microchannels Promote Linear Alignment of Myoblasts

**Figure 4:**
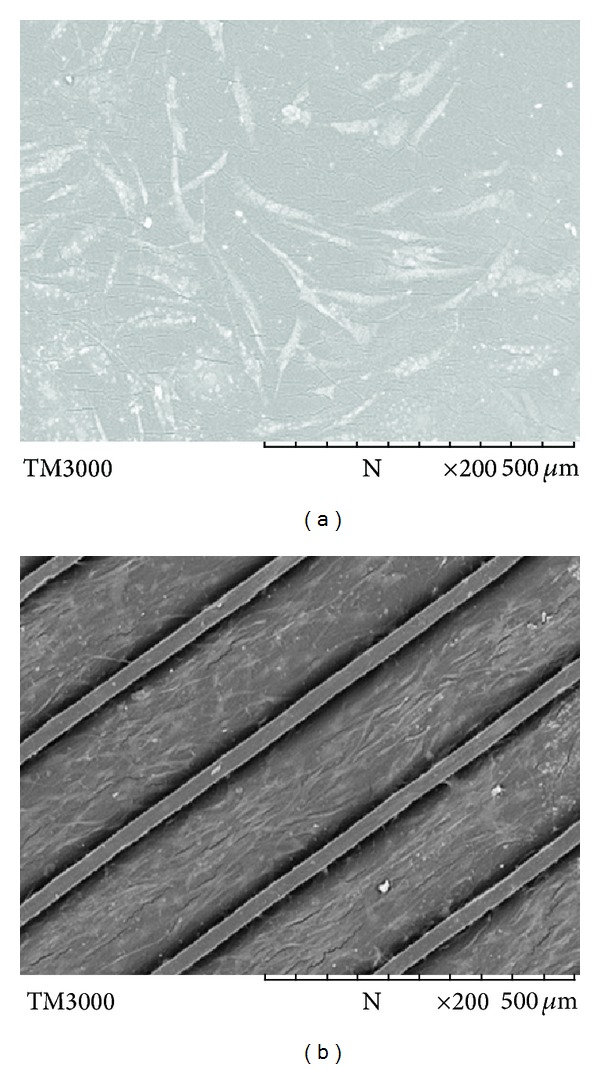
Cell morphology observed under SEM on (a) smooth PU-SF and (b) PU-SF with aligned microchannels. Cells were seeded at the density of 8 × 10^4^ cells mL^−1^ and cultured for 10 days at 37°C in humidified air with 5% CO_2_ (the same conditions were followed for all cell culture).

As the alignment of skeletal muscle cells is an important prerequisite for functional muscle tissue, a polymeric scaffold with unidirectional microchannels was designed to provide environmental cues to mimic the organization of myotubes in hypopharynx skeletal muscle. After 10 days of* in vitro* culture, the seeded myoblasts were found to be aligned parallel to the channels (Figure 4(b)). In contrast, cells cultured on smooth PU surface were found to be in random state (Figure 4(a)). Apart from being an important requirement for functional skeletal muscle tissue, such alignment may also potentially enhance myotube striation by restricting cell spreading to suppress myoblast proliferation and to promote cell fusion and skeletal muscle differentiation.

### 3.3. Effects of Surface Modification on Cell Growth and Differentiation

**Figure 5:**
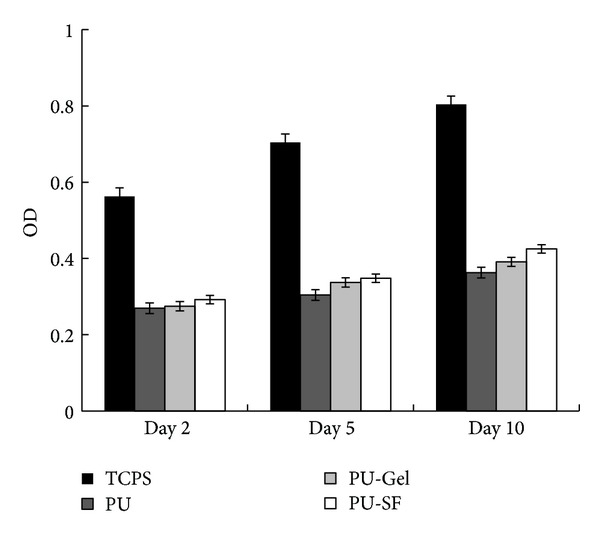
Mitochondrial activity assay: relative absorbance at 490 nm. Cells were seeded at the density of 8 × 10^4^ cells mL^−1^ and cultured for 2, 5, and 10 days, respectively.

Previous reports have demonstrated that surface wettability is primarily influenced by the outermost layer of the polymer, provided that the surface is uniformly flat and can be quantified by measuring the static contact angle of deionized water. Thus, changes in surface wettability resulting from aminolysis and SF or Gel grafting can also be determined by static contact angle measurements. PU surfaces can be modified by techniques including cholesterol [23], plasma [24], photooxidization and ultra-violet (UV) irradiation [25], so that methacrylic acid (MMA), hydroxyethyl acrylate (HEA), acrylic acid, or cholesterol can be grafted. In our work, SF and gelatin were separately grafted onto the PU scaffold surface using the aminolysis and glutaraldehyde cross-linking method [18] because SF and gelatin are nontoxic and have favorable biocompatibility [26, 27]. The grafting of these biomacromolecules resulted in significant increases in wettability of the scaffold surface, compared with that of nonmodified PU surface. The increase in hydrophilicity of both PU-SF and PU-Gel scaffolds enhanced the attachment of myoblasts on scaffolds more than the control PU did (Figure 5, day 2). Thus, both PU-Gel and PU-SF have the higher cell counts (higher Abs) than the unmodified PU, as assessed by the MTT method at day 5 and day 10 (Figure 5), though both of them have still much lower cell counts than TCPS has. It may be caused by the lower attachment area of PU-Gel and PU-SF than smooth TCPS due to the channel-wall patterns. The grafting of biomacromolecules like gelatin and SF greatly improved cell compatibility whereas the intrinsic inert property of PU was comparatively inferior for cell attachment and growth.

**Figure 6:**
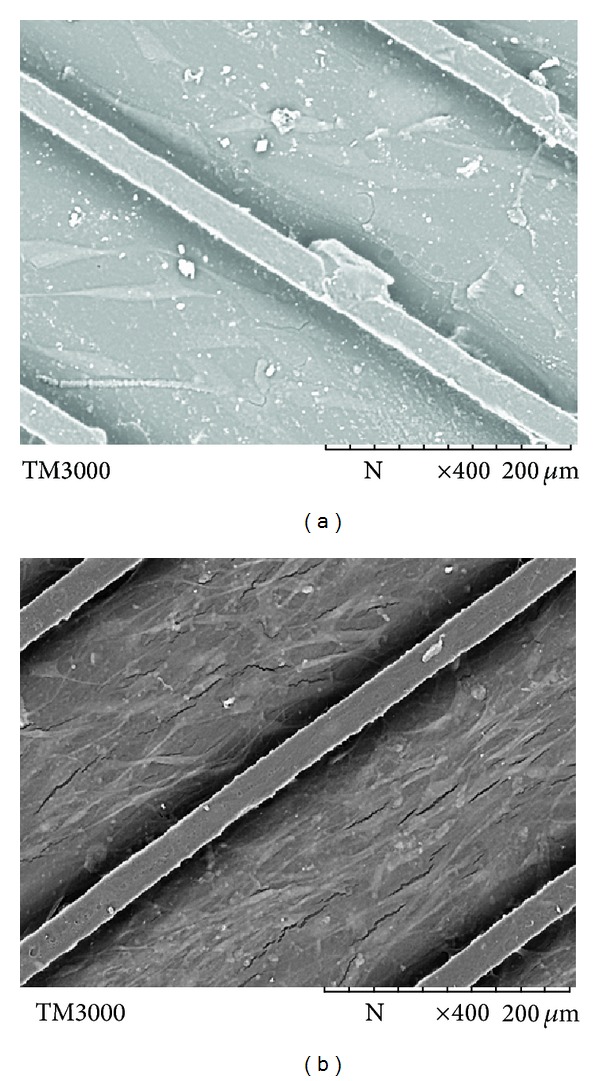
Cell morphology observed under SEM on PU-SF scaffolds with aligned microchannels after (a) 2 days and (b) 10 days.

**Figure 7:**
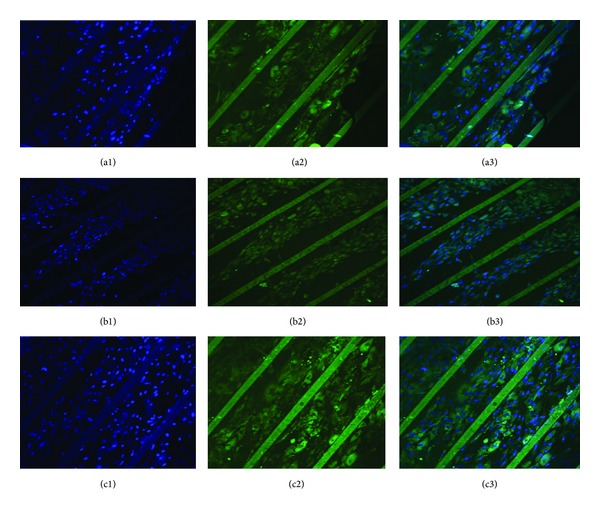
Immunofluorescence staining. Cells were immunostained with primary anti-MyoD1 antibody and anti-mouse IgG (H+L) secondary antibody conjugated with FITC (green, 2). Cell nuclei were stained with DAPI (blue, 1). (3) is the composite of (1) and (2). Cells were seeded at the density of 8 × 10^4^ cells mL^−1^ and cultured for 10 days on three different scaffolds, PU (a): PU-Gel (b), and PU-SF (c).

**Figure 8:**
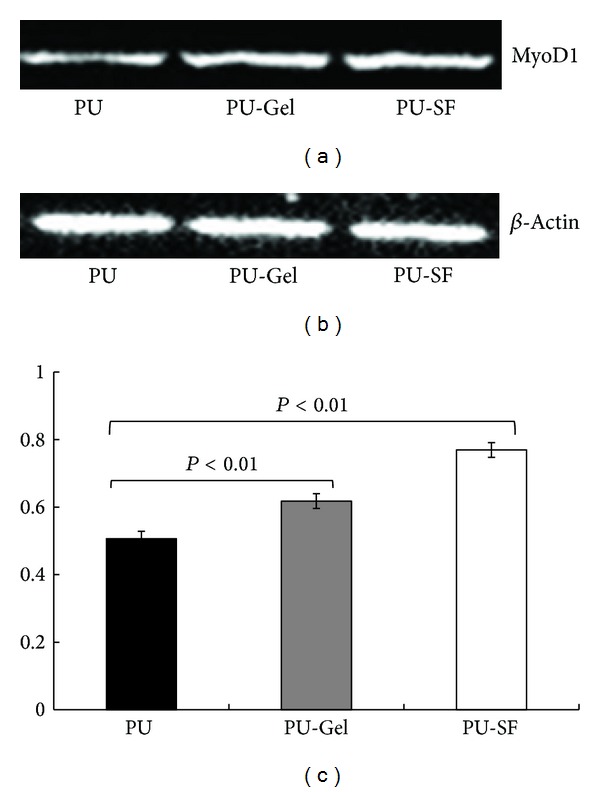
Protein expression of (a) MyoD1 and (b) beta-actin by cells seeded on scaffolds as evaluated by Western blotting. PU, PU-Gel, and PU-SF scaffolds are represented from left to right sequentially, after cells were *in vitro*-cultured for 10 days. (c) is the relative fluorescence density using beta-actin as the reference *P* < 0.01.

As myoblasts grow and subsequently reach confluence, they become slender and spindle-like (Figures 6(a) at day 2 and 6(b) at day 10) [28]. To ascertain the phenotypic state of the myoblasts, immunohistochemistry analysis (Figure 7) and Western blotting (Figure 8) were used for investigation. MyoD1 (myogenic differentiation antigen) is a well-recognized muscle-specific transcription factor expressed in quiescent satellite cells early in the activation process. It also acts as a “master switch” for skeletal muscle differentiation [29]. In response to muscle injury, damage, or degeneration, satellite cells become activated and start to proliferate, giving rise to a population of myogenic progenitor cells known as myoblasts. Myoblasts induce the expression of MyoD1 which is necessary for differentiation into fusion-competent cells. Further fusion into myofibers is associated with an induction of factors essential to myofiber function, including MyoD1, myogenin, and myosin heavy chain (MHC) [29]. The immunofluorescence with anti-MyoD1 as the primary antibody (Figure 7) exhibited that all cells originated from human Hypopharynx grew along the microchannels; the green fluorescence generated from MyoD1 antibody demonstrated that the cells maintained their progenitor capability after they were cultured on scaffolds for 10 days. Figure 8 showed that the MyoD1 expression of cells was related to the surface profiles; cells on PU-SF expressed the highest MyoD1 while the cells on PU expressed the lowest. Thus, PU-SF was considered to be the most favorable choice for PU substrate in the point of skeletal muscle specification. 

## 4. Conclusions

In this study, we have fabricated microchannel patterned scaffold and immobilized proteins like SF and gelatin onto the scaffold surface using aminolysis and glutaraldehyde cross-linking technique. The resulting scaffolds showed the appropriate bulk mechanical properties as well as suitable surface chemistry for cell proliferation and growth. The results about cell culture demonstrated the successful isolation of primary hypopharynx myoblasts, *in vitro* cell expansion, and differentiation into human myofibers using unidirectionally aligned microchannels on PU matrix. We thus concluded the potential of this surface-modified PU scaffolds with aligned microchannels for future applications in skeletal muscle tissue engineering.
